# Study of the biosynthesis and functionality of polyphosphate in *Bifidobacterium longum* KABP042

**DOI:** 10.1038/s41598-023-38082-0

**Published:** 2023-07-08

**Authors:** Cristina Alcántara, Marta Perez, Pol Huedo, Tatiana Altadill, Jordi Espadaler-Mazo, Juan Luis Arqués, Manuel Zúñiga, Vicente Monedero

**Affiliations:** 1 Laboratorio de Bacterias Lácticas y Probióticos, Instituto de Agroquímica y Tecnología de Alimentos (IATA-CSIC), 46980 Paterna, Valencia Spain; 2 R&D Department, AB-Biotics S.A. (Part of Kaneka Corporation), Barcelona, Spain; 3 Basic Sciences Department, Universitat Internacional de Catalunya, Barcelona, Spain; 4 Departamento de Tecnología de Alimentos, Instituto Nacional de Investigación y Tecnología Agraria y Alimentaria (INIA-CSIC), Madrid, Spain

**Keywords:** Applied microbiology, Biotechnology, Microbiology

## Abstract

Polyphosphate (poly-P) biosynthesis in bacteria has been linked to many physiological processes and has been characterized as an interesting functional molecule involved in intestinal homeostasis. We determined the capacity for poly-P production of 18 probiotic strains mainly belonging to *Bifidobacterium* and former *Lactobacillus* genera, showing that poly-P synthesis varied widely between strains and is dependent on the availability of phosphate and the growth phase. Bifidobacteria were especially capable of poly-P synthesis and poly-P kinase (*ppk*) genes were identified in their genomes together with a repertoire of genes involved in phosphate transport and metabolism. In *Bifidobacterium longum* KABP042, the strain we found with highest poly-P production, variations in *ppk* expression were linked to growth conditions and presence of phosphate in the medium. Moreover, the strain produced poly-P in presence of breast milk and lacto-N-tetraose increased the amount of poly-P synthesized. Compared to KABP042 supernatants low in poly-P, exposure of Caco-2 cells to KABP042 supernatants rich in poly-P resulted in decreased epithelial permeability and increased barrier resistance, induction of epithelial protecting factors such as HSP27 and enhanced expression of tight junction protein genes. These results highlight the role of bifidobacteria-derived poly-P as a strain-dependent functional factor acting on epithelial integrity.

## Introduction

Polyphosphate (poly-P) is a linear polymer of inorganic phosphate residues (Pi). This molecule can be synthesized and intracellularly accumulated in form of granules by some microorganisms^1,2^. Accumulating poly-P bacteria (mainly Proteobacteria and Actinobacteria) are used in wastewater plants to remove phosphate^3^. However, poly-P biosynthesis is also an interesting trait in probiotic strains. Probiotics are live microorganisms (mainly lactobacilli and bifidobacteria) that when administered in sufficient amounts have a beneficial effect on the host health^4^. Interestingly, probiotic-derived poly-P was discovered to confer beneficial effects by enhancing the gut barrier function which is key for intestinal homeostasis. In the gut, epithelial cells internalize probiotic-derived poly-P by endocytosis, and poly-P binds to integrin β1 activating p38 MAPK pathway which induces cytoprotective factors such as the heat shock protein HSP27. This induction prevents damage of intestinal barrier^2,5,6^. The role of poly-P in maintaining intestinal homeostasis was confirmed in mice models of colitis where poly-P administration ameliorated inflammation and epithelial injury^5,7–9^, and in a pilot trial in humans, demonstrating clinical remission in patients with refractory ulcerative colitis after poly-P administration^9^.

Thus, poly-P-producing probiotics may have beneficial effects on the human gut by preventing and alleviating intestinal permeability and inflammation. However, the knowledge of poly-P biosynthesis capacity of probiotic species is limited. Some strains of the former genus *Lactobacillus* including *Levilactobacillus brevis* SBC8803^5^, *Lacticaseibacillus casei* BL23^10^, *Lacticaseibacillus paracasei* JCM1163^8^, *Lacticaseibacillus rhamnosus* CRL1505^11^ and *Lactiplantibacillus plantarum* (type strain WCFS1 and others)^2,12^ are known to form poly-P. Some bifidobacterial strains are also suggested to biosynthesize poly-P. *Bifidobacterium longum* ATCC 15707 and the non-probiotic *Bifidobacterium scardovii* BAA-773 strains accumulated granules reminiscent of poly-P^13^; Anand et al.^14^ described strains able to remove phosphate from the medium; and Saiki et al.^8^ measured poly-P in strains of *B*. *longum*, *Bifidobacterium animalis*, *Bifidobacterium breve* and *Bifidobacterium bifidum* species, among others.

For many bacteria poly-P synthesis pathway starts by taking up Pi from the environment through the low-affinity transport system Pit or by the high-affinity ABC-type transporter system PstSCAB. Response to Pi is regulated by two-component regulatory systems (2CRS)^15^. The main enzyme for poly-P synthesis is the poly-P kinase Ppk which forms poly-P from Pi utilizing ATP. Some microorganisms harbor more than one copy of *ppk* gene. Exopolyphosphatases (Ppx or Ppx-GppA) hydrolyze poly-P chains and in some actinobacterial species poly-P-dependent glucokinases Ppgk use poly-P as donor for glucose phosphorylation^3,10^. These routes are less studied in probiotic species. Species of the lactobacilli group harbor a *ppk* gene flanked by two *ppx* genes^10^, while bifidobacteria carry the conserved 2CRS PhoRP that controls Pi response^15^, and *ppk* gene has been detected in *B. scardovii*^13^ (Supplementary Fig. 1).

Poly-P concentration usually shows an increase during logarithmic growth phase, then decreases gradually up to the stationary phase and can reach undetectable levels. Although poly-P is not actively transported, some levels can be detected extracellularly^8^. The physiological role of poly-P remains unclear and it has been linked to stress responses such as oxidative^10,13^, acidic^10,13,16^ or osmotic stress conditions^10^. It is known that Pi concentration in the medium affect poly-P formation in lactobacilli^10^. Carbon and nitrogen sources are suggested to affect phosphate removal in bifidobacteria^14^ and polyamines (putrescine, spermine and spermidine) increase poly-P accumulation in *Escherichia coli*^17^. However, the effect of these and other factors is less understood.

The beneficial role of several probiotic strains is linked to the protection of the intestinal barrier^18^. However, whether this effect involves the biosynthesis and action of poly-P is not well known. In this work, we studied the poly-P production capacity of 11 distinct species of probiotics and deepened in bifidobacteria group analyzing poly-P-related genes presence and organization in genomes. In addition, we used the poly-P-producer probiotic strain *B. longum* KABP042 to investigate the effect of environmental Pi. Since KABP042 was isolated from the faeces of a breastfed infant^19^ and breast milk contains Pi^20^, the effect of breast milk and milk bioactive compounds including human milk oligosaccharides (HMO) and polyamines on poly-P synthesis was also investigated. Furthermore, the effect of *B. longum* KABP042-derived poly-P in the human intestinal barrier was assessed in vitro by measuring barrier permeability and resistance and the induction of protective factors involved in barrier integrity.

## Results

### Poly-P biosynthesis by probiotic strains

Poly-P biosynthesis capacity of 18 strains belonging to 11 different probiotic species was assessed by measuring intracellular poly-P accumulation in exponential and stationary phases of the growth curve in MEI or MRSc media. In total, we evaluated 13 probiotic strains belonging to the bacterial species *B. animalis* (1), *B. bifidum* (1), *B. breve* (2), *B. longum* (4), *L. rhamnosus* (1), *L. plantarum* (1), *Limosilactobacillus reuteri* (1), *Pediococcus pentosaceus* (1) and the yeast *Saccharomyces boulardii* (1). Five strains with published evidence of poly-P metabolism were included as controls for comparison: *B. breve* JMC1273, *B. longum* ATCC15707, *B. scardovii* BAA773, *L. paracasei* JMC1163 and *L. plantarum* WCFS1 (Table 1).

**Table 1:** Strains used in this study.

Strain	Source	Classification	References
*Bifidobacterium animalis* BB12	Commercial product	Probiotic	^21^
*Bifidobacterium bifidum* P671	INIA-CSIC	Probiotic	^22^
*Bifidobacterium breve* JCM1273 (DSM20091)	DSM	Control	^23^
*Bifidobacterium breve* M16V	Commercial product	Probiotic	^24^
*Bifidobacterium breve* P734	INIA-CSIC	Probiotic	^22^
*Bifidobacterium longum* subsp*. longum* 35624	Commercial product	Probiotic	^25^
*Bifidobacterium longum* subsp*. longum* ATCC15707	ATCC	Control	^13^
*Bifidobacterium longum* subsp*. longum* BB536	Commercial product	Probiotic	^26^
*Bifidobacterium longum* subsp*. longum* KABP042 (CECT7894)	AB-Biotics S.A.	Probiotic	^19^
*Bifidobacterium longum* subsp*. longum* P132	INIA-CSIC	Probiotic	^27^
*Bifidobacterium scardovii* BAA773 (DSM13734)	DSM	Control	^13^
*Lacticaseibacillus paracasei* JCM1163 (DSM20006)	DSM	Control	^8^
*Lacticaseibacillus rhamnosus* GG (ATCC53103)	ATCC	Probiotic	^28^
*Lactiplantibacillus plantarum* 299v	Commercial product	Probiotic	^29^
*Lactiplantibacillus plantarum* WCFS1 (ATCC BAA-793)	ATCC	Control	^2^
*Limosilactobacillus reuteri* DSM17938	Commercial product	Probiotic	^30^
*Pediococcus pentosaceus* KABP041 (CECT8330)	AB-Biotics S.A	Probiotic	^19^
*Saccharomyces boulardii* CNCM I-745	Commercial product	Probiotic	^31^

References of probiotic effects for probiotic strains or poly-P metabolism for control strains are indicated. Routine growth conditions were: MRS supplemented with 0.1% (w/v) cysteine-HCl (MRSc) and anaerobiosis for bifidobacterial strains; MRS medium and anaerobiosis for lactobacilli and *P. pentosaceus* strains; YDP medium and aerobiosis for *S. boulardii* strain. All strains were grown at 37 °C.

CECT, Spanish Type Culture Collection; ATCC, American Type Culture Collection; DSM, German Collection of Microorganisms and Cell Cultures; INIA-CSIC, National Institute for Agricultural and Food Research and Technology (Spain).

**Figure 1:**
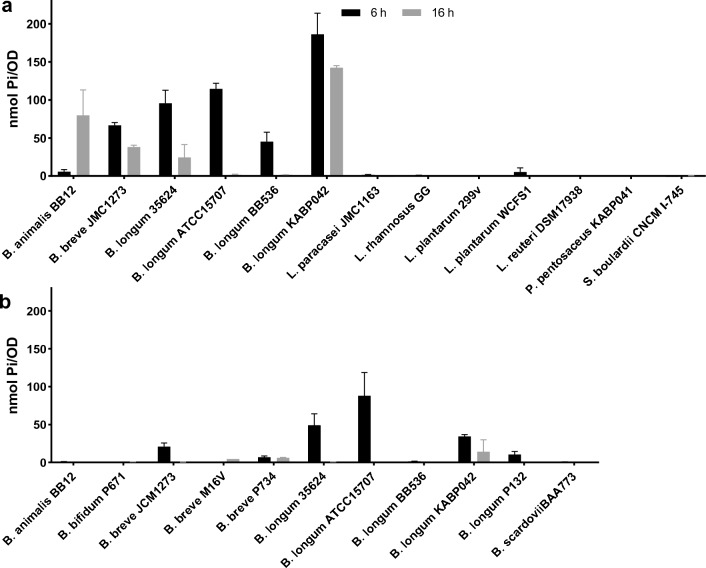
Poly-P accumulation in the studied strains after 6 and 16 h of growth in (**a**) MEI medium and (**b**) MRSc medium.

Strains were first cultured in MEI medium which allows a higher poly-P formation compared with MRSc probably due to its high concentration of phosphate^10^. However, some bifidobacteria strains failed to grow in this medium and were cultured in MRSc instead. Bifidobacteria strains able to grow in MEI were also cultured in MRSc for comparison (Fig. 1). OD values varied between strains (Supplementary Table S1) and thus poly-P amounts were normalized to OD. Nevertheless, strain comparisons were made between the strains grown in the same medium.

In general, bifidobacteria strains accumulated greater amounts of poly-P than lactobacilli strains while poly-P detected in *P. pentosaceus* and *S. boulardii* was very low (Fig. 1a and Supplementary Table S1). Comparisons of poly-P levels in strains that reached similar OD values growing in the same medium (Supplementary Table S1) showed poly-P biosynthesis varied greatly between strains. Levels were generally higher at 6 h than 16 h of growth except for *B. animalis* BB12 and *B. breve* M16V which accumulated more at 16 h (Fig. 1a,b), indicating poly-P accumulation fluctuates with the growth curve.

Among strains of the former *Lactobacillus* genus, control strains *L. paracasei* JMC1163 and *L. plantarum* WCFS1 showed the greater poly-P values while the other strains accumulated very low amounts (Fig. 1a and Supplementary Table S1). All bifidobacteria strains were able to accumulate some poly-P amount but the quantity was very low in *B. bifidum* P671 and the control strain *B. scardovii* BAA773 (Fig. 1b). Notably, *B. longum* strains showed the greater poly-P biosynthesis capacity, suggesting that this trait is conserved in the species. Interestingly, *B. longum* KABP042 accumulated the highest levels at both time-points and was the only strain showing this profile. As expected, levels of poly-P were generally lower in MRSc than MEI medium for those bifidobacteria able to grow in both media, despite they reached higher OD values in MRSc (Fig. 1a,b and Supplementary Table S1). These outcomes confirm MEI medium permits a greater poly-P synthesis and strains can biosynthesize poly-P with high capacity despite showing low growth.

In silico search of poly-P kinase *ppk* gene was carried out in the available genomes of the studied strains. Gene *ppk* was not found in *P. pentosaceus* KABP041 strain which concordantly showed very low poly-P levels. Consistently with phenotypic results, all bifidobacterial genomes harbor the gene, suggesting it is a spread trait among bifidobacteria (Table 2).

**Table 2:** Identification of *ppk* gene in the available genomes of studied strains by BLAST.

Strain	Genome accession no.	Reference genome	*ppk* identity (%)
*B. animalis* BB12	NC_017214.2	AE014295.3	82
*B. bifidum* P671	JAPFGE000000000	AE014295.3	84
*B. breve* P734	NZ_CABFNK000000000.1	AE014295.3	93
*B****.**** longum* 35624	CP013673.1	AE014295.3	99
*B. longum* ATCC15707	GCA_000196555.1	AE014295.3	98
*B****.**** longum* KABP042	JAHTKJ000000000.1	AE014295.3	99
*B. scardovii* BAA773	AP012331.1	AE014295.3	85
*L. rhamnosus* GG	FM179322.1	NC_004567.2	64
*L. plantarum* WCFS1	NC_004567.2	NC_004567.2	100
*P. pentosaceus* KABP041	JAHTMM000000000.1	NC_004567.2	ND

Genomes were obtained from NCBI except ATCC15707 genome which was obtained from the American Type Culture Collection (ATCC). Genomes not included in the analysis were not available in any public database. Percent identity was calculated against reference genomes: *B. longum* NCC2705 for bifidobacterial *ppk* and *L. plantarum* WCSF1 for lactobacilli *ppk.*

*ND* not detected.

### Organization of poly-P-related genes in bifidobacteria

We further investigated the presence of other poly-P-related genes and their organization in the genomes of bifidobacterial strains. Nucleotide sequences of poly-P-related genes of *B. longum* BXY01 were used as template in the in silico analyses because this genome contains all genes of interest. These included the two-component regulatory system (2CRS) *phoRP*, the high-affinity phosphate ABC-type transporter *pstSCAB*, the low-affinity Pi-specific transporter *pit*, the poly-P-dependent glucokinase *ppgk*, the exopolyphosphatase *ppx-gppA* and the poly-P kinase *ppk*^3,15^.

**Figure 2:**
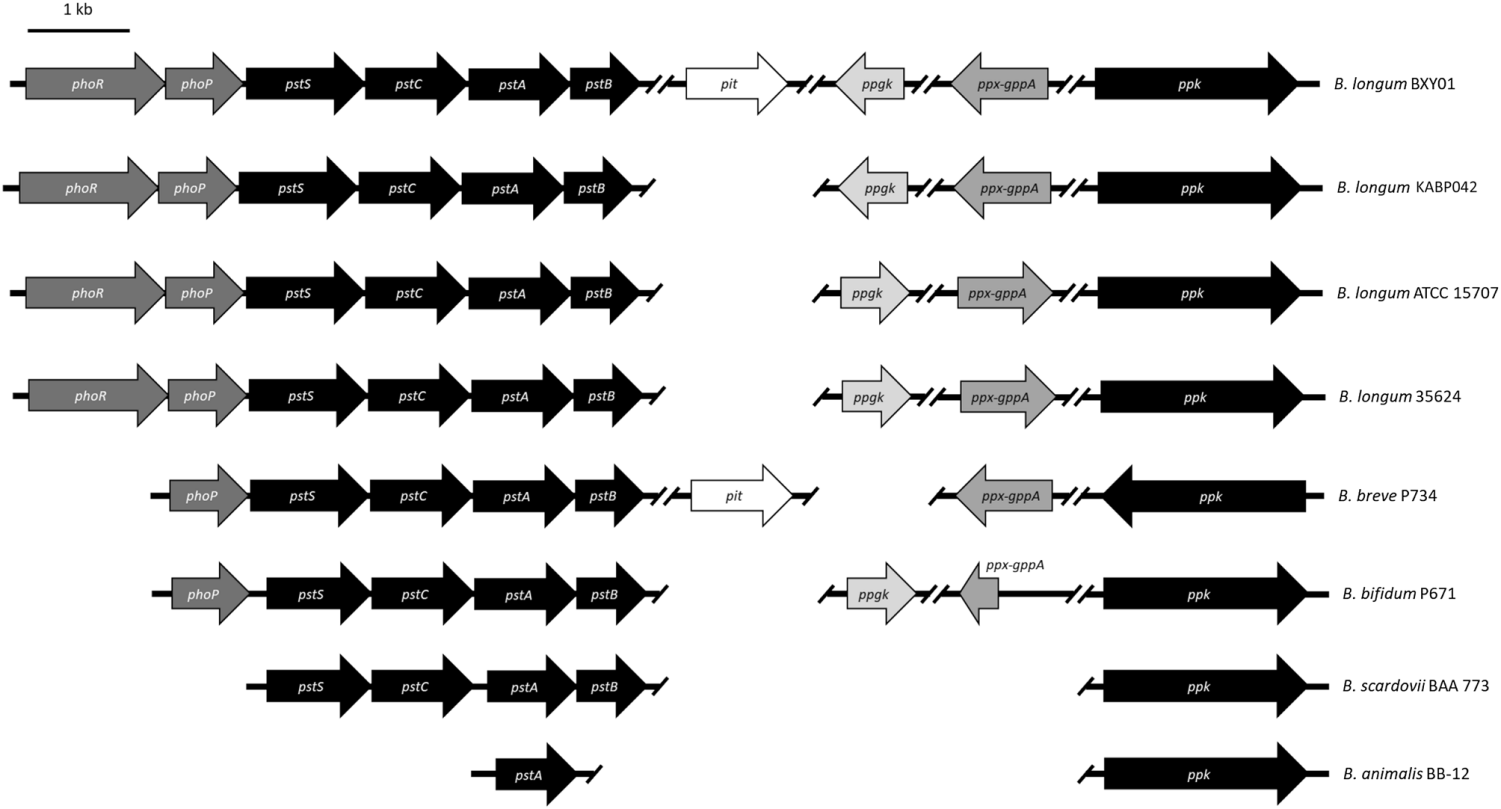
Schematic illustration of the presence and distribution of the *phoRP, pstSCAB, pit, ppgk, ppx-gppA* and *ppk* genes in studied *Bifidobacterium* spp strains. Each solid arrow indicates an open reading frame (ORF). The lengths of the arrows are proportional to the length of the predicted ORF.

As shown in Fig. 2 and Table 2, all bifidobacterial strains investigated harbored the poly-P kinase *ppk* gene, but the distribution of the rest of associated genes varied greatly between strains. Similar to *B. longum* BXY01, *B. longum* strains KABP042, ATCC15707 and 35624 displayed almost identical gene repertoire except for the low-affinity phosphate transporter *pit*, which was absent in their genomes. Genome of *B. breve* P734 contained a truncated 2CRS lacking the membrane-associated histidine protein kinase gene *phoR*. This genome also lacked the poly-P-dependent glucokinase *ppgk*, but one copy of the transport protein *pit* was detected. In the genome of *B. bifidum* P671 the membrane-associated histidine kinase *phoR* and the phosphate transporter *pit* were not identified, and the exopolyphosphatase gene *ppx-gppA* was truncated. In the genome of *B. scardovii* BAA773, only the high-affinity ABC-type transporter *pstSCAB* was detected, apart from the *ppk* gene. Finally, only the *pstA* and the *ppk* genes were identified in the genome of *B. animalis* BB12.

### Synthesis of poly-P and *ppk* expression in *B. longum* KAPB042

**Figure 3:**
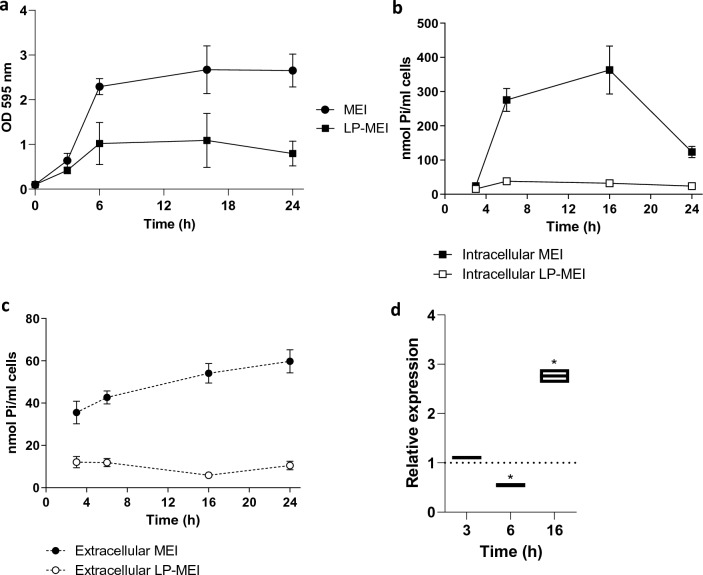
Poly-P production in *B. longum* KABP042. (**a**) Growth in MEI and LP-MEI. (**b**) Levels of intracellular and (**c**) extracellular poly-P as a function of growth. The amount of poly-P (as nmol Pi) from bacteria present in one ml of culture volume and nmol Pi/ml of cell-free culture supernatant are depicted, respectively. Two-way ANOVA with Sidak's multiple comparisons test was employed (see text for *p* values). (**d**) Expression of *ppk* gene (3, 6 and 16 h from panel a) determined by qPCR. Data represent fold changes relative to reference conditions (growth in LP-MEI). The Pair Wise Fixed Reallocation Randomisation Test implemented in REST was employed and asterisks indicate *p* < 0.0001.

Since *B. longum* KAPB042 was the highest poly-P producer within the probiotics tested, we decided to study its poly-P synthesis capacity in more detail. Growth and poly-P accumulation in this strain was assayed under high (MEI medium) or low Pi conditions (LP-MEI medium). Growth attained under low Pi was lower (*p* < 0.05 for 6, 16 and 24 h; Fig. 3a). The amount of poly-P accumulated inside the cells was higher under high Pi compared to low Pi conditions and it suffered a reduction at late stationary phase (*p* < 0.001 for 6, 16 and 24 h; Fig. 3b). It was also observed that the amount of poly-P in growth supernatants was low compared to intracellular poly-P, showing that a small proportion of synthesized poly-P is excreted outside the bacteria (Fig. 3c). Poly-P amounts were greater in supernatants from MEI cultures than LP-MEI mirroring the profile inside the cells (*p* < 0.01 for 16 and 24 h; Fig. 3c). Expression of *ppk* in *B. longum* cells was higher for cells grown in MEI compared to LP-MEI at late growth phase (16 h, *p* < 0.001; Fig. 3d), while differences at other time points of the growth curve were low, suggesting a relative impact of Pi availability in the modulation of *ppk* expression. In addition, we confirmed the presence of poly-P granules in these cells, which showed the characteristic intracellular inclusions after specific staining. A visual estimation of granule numbers and density showed that they were also higher in MEI compared to LP-MEI (Supplementary Fig. 2).

### *B. longum* KAPB042 biosynthesizes poly-P in presence of breast milk and accumulation is affected by the consumed HMO LNT but not by polyamines

*B. longum* is a typical inhabitant of the intestinal tract of breast-fed infants. Specifically, the strain KABP042 originated from feces of a breastfed infant and is known to exert beneficial effects in lactating infants with colic^19^. Because *B. longum* and human milk interact in the infant gut we tested the effect of breast milk (which is known to contain around 4.5 mM Pi^20^) and specific milk components on poly-P synthesis in the KAPB042 strain. Previous studies have suggested carbon source and polyamines can impact bacterial poly-P metabolism^14,17^. Thus we decided to study the effect of human milk oligosaccharides (HMO) which are known to be specifically consumed by some bifidobacteria^32^ and the effect of polyamines at levels found in breast milk. Since there are over 200 different HMO structures in human milk^32^, we first studied the HMO-metabolizing capacity of *B. longum* KABP042.

**Figure 4:**
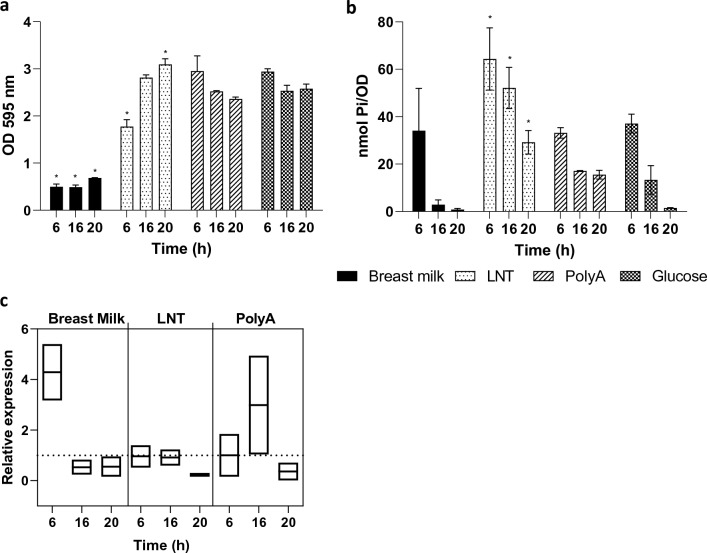
Effect of different components of the growth medium in poly-P synthesis in *B. longum* KABP042. (**a**) Growth in MEI medium with different carbon sources (glucose and LNT), human milk added at 1% v/v (no glucose added) and MEI plus a mixture of polyamines (polyA). (**b**) Poly-P synthesis in the conditions shown in a. Asterisks indicate a significant difference with *p* < 0.01 (Two-way ANOVA, Dunnett multiple comparisons test) with respect to glucose. (**c**) *ppk* expression determined by qPCR. Relative expression is expressed as fold changes in gene expression using growth in MEI (with glucose) as a reference.

The genome of *B. longum* KABP042 was first screened for the presence of carbohydrate metabolizing enzymes through eggNOG mapper^33^ and dbCAN annotation tools^34^. In silico analyses revealed the presence of 86 Carbohydrate Active enZYmes (CAZy), of which 58 were glycoside hydrolases (GH), and 26 glycosyl transferases (GT). Among GH enzymes, most common families were GH43 and GH13, which are involved in the degradation of a wide range of carbohydrates, including plant-derived polysaccharides^35,36^. Further analyses indicated KABP042 harbors genes encoding HMO-degrading enzymes including the *lnbXY* locus (lacto-N-biosidase), which specifically degrades the HMO lacto-N-tetraose (LNT)^37^, in addition to beta-galactosidase, alpha-galactosidase and beta-glucuronidase genes. In this line, in vitro experiments showed the strain grows in medium with LNT as unique carbon source (Supplementary Fig. 3), confirming *B. longum* KABP042 can utilize the HMO LNT. Therefore, we studied the effect of this specific HMO on poly-P biosynthesis. The ability to synthesize poly-P in KABP042 was determined in medium MEI supplemented with different carbon sources (human milk, LNT or glucose as control) and in MEI containing the polyamines putrescine, spermidine and spermine. Although growth in the presence of human milk was low under our experimental conditions (*p* < 0.0001 for all time points), the levels of poly-P were similar to those obtained with glucose during the first stage of growth (Fig. 4a,b). Growth in MEI with LNT as carbon source was lower compared to glucose (*p* < 0.0001 at 6 h; Fig. 4a), but it resulted in high contents of intracellular poly-P, that remained for a longer period (*p* = 0.008, 0.0006 and 0.007 for 6, 16 and 20 h, respectively; Fig. 4b). The presence of polyamines in MEI had low effects on the maximal amount of poly-P, although their levels diminished slower compared to control. In these new experiments the amount of poly-P detected in KABP042 was noticeably lower compared to the previous measurements. We have noticed this effect when batches of complex MEI components were renewed, suggesting a strong effect of medium composition on poly-P yields. The expression of *ppk* was measured under these conditions (Fig. 4c). Compared to growth in MEI with glucose, growth with added breast milk resulted in enhanced *ppk* expression during early exponential phase concordantly with poly-P production, while the presence of polyamines resulted in increased expression at 16 h but these changes did not reach statistical significance LNT did not affect *ppk* expression despite the observed differences in poly-P biosynthesis. No differences were observed in late stationary cells between the different growth conditions. These outcomes suggest *B. longum* KABP942 may synthesize poly-P in presence of breast milk and the HMO LNT enhances the production. Although some breast milk components may affect *ppk* expression other post-transcriptional regulatory mechanisms seem to be involved in poly-P metabolism.

### Poly-P derived from *B. longum* KABP04 increases the integrity of the intestinal barrier and reduces permeability by upregulating the production of HSP27 and the expression of tight junction proteins

**Figure 5:**
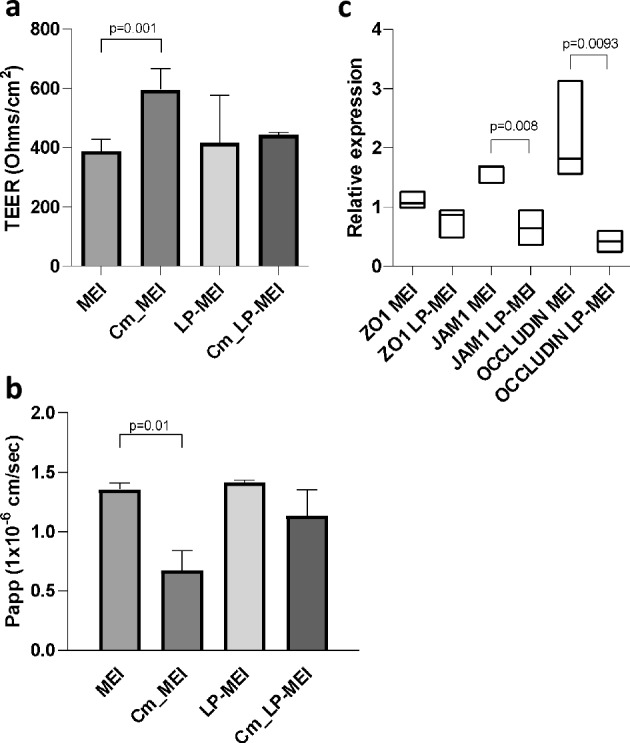
Effects of *B. longum* KABP042 conditioned media on intestinal permeability in Caco-2 model. (**a**) Transepithelial electrical resistance (TEER) in Caco-2 exposed to conditioned MEI or LP-MEI (Cm; medium fermented by KABP042, filtered and neutralized), respectively, for 72 h. (**b**) Apparent permeability (P_aap_) to Lucifer Yellow in Caco-2 cells monolayers exposed to MEI and LP-MEI conditioned or non-conditioned media for 72 h. One-way ANOVA with Sidak's multiple comparisons test was employed. (c) Relative expression of the genes (fold change) for tight junction proteins (ZO-1, OCLN, JAM-1) determined by qPCR. The reference conditions were those of cells cultivated in the presence of non-conditioned MEI or LP-MEI, respectively. The Pair Wise Fixed Reallocation Randomisation Test implemented in REST was employed.

We tested the role of *B. longum-*derived poly-P in intestinal homeostasis by measuring whether Caco-2 cells monolayers exposed to neutralized supernatants of *B. longum* KABP042, at the apical side of Transwell inserts, presented changes in the functionality of the cellular barrier. To this end, supernatants of bacteria grown under high or low Pi conditions were used. As can be seen in Fig. 5a, the TEER of Caco-2 monolayers exposed to *B. longum* KABP042 growth supernatants (10% v/v; conditioned medium) carrying high poly-P (grown in MEI, Fig. 3b) was significantly higher compared to culture supernatants with low poly-P contents (grown in LP-MEI) (*p* = 0.001). Consistently, permeability (*P*_*app*_) to Lucifer Yellow dye was significantly lower in cells exposed to MEI conditioned by *B. longum* KABP042 (Fig. 5b) compared to conditioned LP-MEI (*p* = 0.01). These outcomes suggest poly-P produced by *B. longum* KABP042 can exert a protective effect on the gut barrier by increasing resistance and reducing permeability of the monolayer.

**Figure 6:**
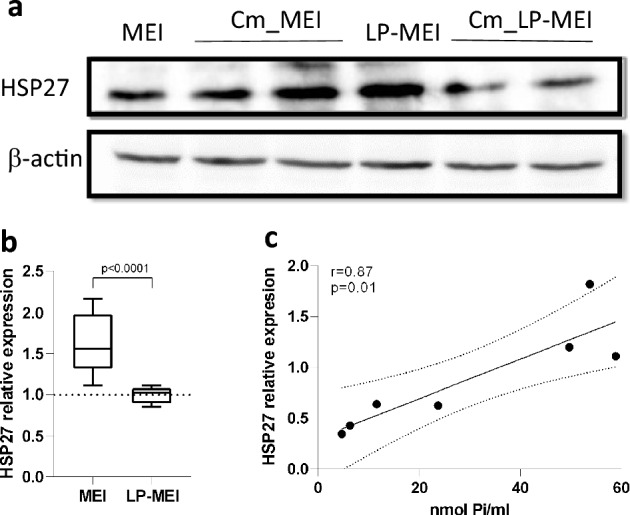
Induction of HSP27 in Caco-2 cells by incubation with supernatants of *B. longum* KABP042. (**a**) Western blot with anti-HSP27; MEI and LP-MEI are non-fermented media; Cm, conditioned medium (i.e. fermented by KABP042, filtered and neutralized); β-actin was used as a loading control. Two representative samples of Caco-2 cells treated with Cm are seen. The gels were transferred and the membranes were cut in two pieces that were probed with anti-HSP27 or anti-β-actin, respectively. Complete gel images are provided in Supplementary Information. (**b**) Comparison of the relative expression of HSP27 between Caco-2 cells exposed to MEI or LP-MEI conditioned media. In each case the expression is normalized to that of the corresponding non-conditioned medium. Student's t-test was applied. Lowest expression was set to 1, n ≥ 3. (**c**) Correlation between the amount of poly-P in *B. longum* KABP042 supernatants and the relative expression of HSP27 in Caco-2 cells.

In order to investigate the mechanisms involved in the protection of the intestinal epithelium by *B. longum* KABP042-derived poly-P, we measured expression of genes encoding proteins from the tight junctions (ZO1, JAM1 and OCLN) that are crucial for the maintenance of the barrier integrity^38^. The presence of conditioned MEI medium resulted in higher gene expression for JAM-1 (*p* = 0.008) and OCLN (*p* = 0.0093) in Caco-2 cells (Fig. 5c), indicative of a stronger functional barrier. We also determined the levels of HSP27, which is known to be modulated by poly-P produced by some lactobacilli^5^. The intestinal epithelial cell line Caco-2 was exposed to *B. longum* KABP042 growth supernatants from cultures in MEI and LP-MEI. Addition of fresh MEI or LP-MEI to the cultures (control) showed no differences in HSP27 induction; however, conditioned MEI or LP-MEI showed differences in HSP27 levels, with MEI supernatants displaying a significantly higher HSP27 relative expression (*p* < 0.0001) (Fig. 6a,b). Furthermore, a significant correlation was also observed between HSP27 expression and poly-P amounts in supernatants of *B. longum* (Pearson r = 0.87, *p* = 0.01) (Fig. 6c).

## Discussion

The study of poly-P production in lactobacilli, bifidobacteria and probiotics in general received low attention until this molecule was reported as a functional bacterial metabolite^10^. Since then, probiotic-derived poly-P has been characterized as a beneficial molecule that protects against intestinal injury^7,9^, but additional functions in preventing inflammatory processes at different locations are also being reported^39,40^. In this study, we show that, under the tested conditions with our collection of probiotics, production of poly-P was highly variable among strains. Notwithstanding strain variability, bifidobacteria, especially *B. longum* subsp. *longum*, were among the best producers.

As occurred with lactobacilli^10^, poly-P production in bifidobacteria was linked to the presence of *ppk* gene. In bifidobacteria, *ppk* and *ppx* genes do not form an operon, contrarily to what has been reported for lactobacilli^1,10^. The presence of elevated amounts of Pi was the principal factor affecting poly-P accumulation in the assayed strains, as previously showed in lactobacilli^10^. A repertoire of genes involved in Pi uptake (*pst* and *pit* genes) was also found in bifidobacteria, a factor known to influence phosphate incorporation in other bacteria^41,42^. However, it is not known at this stage whether the presence of such complement of genes has any impact on the capacity of a certain *Bifidobacterium* strain to accumulate poly-P. Our results indicate that certain variability exists within *Bifidobacterium* spp. in relation to the distribution of these poly-P-related genes, especially the *pit* gene, which was only detected in some *B. longum* and *B. breve* strains, highlighting that some traits are species- and strain-specific. *B. longum* strains display the maximum number of poly-P-associated genes. These observations were in line with the in vitro experiments, since *B. longum* strains were those producing greater levels of poly-P. On the contrary, *B. animalis* BB12 also accumulated high levels of poly-P despite harboring the lesser number of these genes. Variation in poly-P levels and genes between strains suggest different regulation mechanisms must be involved in poly-P biosynthesis. Further research should be conducted to determine the role of these and putative additional genes in the poly-P synthesis and accumulation in bifidobacteria.

Besides of Pi availability in the medium, the conditions that govern poly-P levels in bifidobacteria are unclear. The sharp drop of intracellular poly-P levels at the onset of stationary phase in *L. paracasei* JCM 1163^8^ suggests that poly-P plays an active role during the physiology of stationary cells. In the case of *B. longum* KABP042 poly-P concentration remained high during early stationary phase. It is postulated that poly-P levels in cells are governed by the opposite activities of Ppk (poly-P forming) and Ppx (poly-P degrading) enzymes^43^, while gene regulation would have a minor impact (as mentioned, *ppk* and *ppx* are cotranscribed in a large number of bacteria). However, we showed that in *B. longum* KABP042 expression of *ppk* was favored by growth with high levels of Pi at early stationary phase, but it also suffered variations depending on other growth conditions, such as the presence of human milk. On the contrary, although consumption of the HMO LNT by *B. longum* KABP042 increased the biosynthesis of poly-P, the expression of *ppk* was not affected indicating other regulatory mechanisms are involved. Further studies are warranted to understand this result as it represents an interesting synergic effect for breasted infants administered with this probiotic strain*.* Although Anand et al.^23^ have suggested that carbon sources such as fructooligosaccharides influence phosphate metabolism in bifidobacteria, this is the first time that the effect of breast milk and HMO in poly-P biosynthesis is investigated. In this regard, HMO consumption by bifidobacteria has sometimes been treated as a desirable trait per se to facilitate intestinal colonization, but may in fact be just a means to an end: the actual production of bacterial biomolecules supporting the homeostasis of the host.

The maintenance of intestinal barrier integrity is one of the many attributes of probiotics and the interaction with several cellular pathways has been evidenced for this effect^44^. Poly-P has emerged as a functional molecule produced by probiotics which acts in this sense^5,6,8^. We showed that, as reported in other strains^2,5^, supernatants of *B. longum* KABP042 conferred strengthening of the intestinal barrier in the Caco-2 cell model by upregulating HSP27. At the same time, the expression of tight junction proteins genes was affected, which most probably results in the reduced permeability that was found in this model when exposed to *B. longum* KABP042 supernatants. Of note, reduced permeability was not observed by treating Caco-2 cells with fresh MEI medium, indicating the effect is not due simply to high Pi content. Poly-P derived from *L. paracasei* JCM 1163 was also shown to reduce permeability in mice small intestine explants exposed to oxidants^8^ and the mechanism for this was hypothesized to be dependent on TNFAIP3 induction, a factor involved in cytokine-mediated immune and inflammatory responses and which increases intestinal barrier function via modulation of tight junction proteins^6^.

The differences that we observed in Caco-2 cells after incubation with conditioned MEI or LP-MEI supernatants were most probably linked to differences in poly-P levels between both cultures. However, strains unable to synthesize poly-P are needed to ascertain this point, as it cannot be ruled out that other components from bifidobacteria were having also an effect. Such knockout mutants in the *ppk* gene were obtained for two strains of *L. plantarum,* where the involvement of poly-P synthesis in HSP27 induction was verified^2^. The variations observed while using growth supernatants with high or low Pi implies that the growth conditions or the environment where the probiotic develops will have an impact on its functional properties, as already reported^45,46^. In fact, we showed that in *B. longum* KABP042, isolated from breastfed infant feces, the presence of breast milk or other human milk components such as LNT impacted poly-P yields.

Some potential limitations for the application of poly-P producing bifidobacteria are the fluctuations in the levels of the polymer synthesis, and the fact that poly-P is a cytoplasmic molecule in bacteria, but its beneficial activity seems to require extracellular presence. Our results as well as those from a previous publication with mutant strains from *L. paracasei* JCM 1163 indicate that extracellular poly-P represents a low proportion of the polymer found inside the cells^8^. Poly-P is most probably not actively excreted to the growth medium and its presence outside the cells could be a mere effect of cell leakage, cell lysis or poly-P present as part of extracellular vesicles^44^. Accordingly, higher intracellular accumulation would be expected to also result in higher extracellular levels, even if the latter remain a fraction of the former. In this regard, our results show that accumulation kinetics of extracellular poly-P grows across time, possibly following accumulation of intracellular one. Of note, even the low extracellular poly-P levels produced by KABP042, compared to intracellular amounts, were probably one of the factors exerting a significant effect in the gut barrier, as demonstrated in our experiments. Interestingly, we observed a decrease of intracellular poly-P at the late stationary phase. The transitory accumulation of Poly-P along the growth curve is described in other bacteria^8^, however, the reasons of this behavior are unknown. Nevertheless, screening of bifidobacterial strains with high poly-P synthesis and, if possible, increased poly-P excretion, could constitute a valuable strategy to study poly-P functionality and to obtain new candidate probiotics.

In conclusion, we showed that probiotic bifidobacteria may be good producers of poly-P, although the factors and growth conditions that trigger this production still need to be more clearly determined. Bifidobacterial-derived poly-P represents a probiotic factor with potential to protect the epithelial integrity and its synthesis could be included in strain screening and selection processes.

## Methods

### Strains and culture conditions

Strains used in this study and their sources are indicated in Table 1. To isolate strains from commercial products, capsule content was resuspended in medium and plated on agar plates. Single colonies were obtained and grown to prepare glycerol stocks. Species identity was confirmed by PCR amplification and sequencing of the 16S rRNA gene.

Routine medium was MRS for lactobacilli and *Pediococcus pentosaceus*, MRS supplemented with 0.1% (w/v) cysteine-HCl (MRSc) for bifidobacteria, and YDP medium for *Saccharomyces boulardii*. The strains were cultured at 37 °C under anaerobic conditions except *S. boulardii* which was cultured aerobically. For poly-P production assay, MEI medium was used^2^ (per liter: 0.5% yeast extract, 0.5% tryptone, 0.4% K_2_HPO_4_, 0.5% KH_2_PO_4_, 0.02% MgSO_4_·7H_2_O, 0.005% MnSO_4_, 0.05% cysteine, and 0.5% glucose (w/v) and 1 ml of Tween 80). A low-phosphate MEI medium (LP-MEI) was formulated without the addition of K_2_HPO_4_, and KH_2_PO_4_.

### In silico analysis

Nucleotide sequences for *ppk* gene were retrieved from the NCBI from reference genomes AE014295.3 (*B. longum* NCC2705) and AL935263.2 (*L. plantarum* WCFS1) respectively, and subjected to BLAST analysis^47^ against available genomes of studied strains. Genome accession numbers are indicated in Table 1. To study other polyP-related genes and their organization in bifidobacterial strains, genome sequence of *B. longum* BXY01 was retrieved from the NCBI (accession number CP008885.1) and nucleotide sequences of genes *phoR, phoP, pstS, pstC, pstA, pstB, pit, ppgK, ppx-ggpA* and *ppk* were used as reference. Genome sequences of *Bifidobacterium* spp. strains were inspected for the presence of polyP-related genes using BLAST^47^.

To identify genes related to carbohydrate metabolism including HMOs, the genome of *B. longum* KABP042 was annotated using eggNOG v.4.5.1 platform^33^ and the specific detection of carbohydrate active enzymes was performed using the dbCAN automated CAZy annotation meta server^34^.

### Polyphosphate granules staining

Observation of poly-P granules accumulated in *B. longum* KABP042 as a function of growth was performed by harvesting the bacteria after growing them in 15 ml of MEI or LP-MEI medium at 37 °C under anaerobic conditions at different points of the growth curve (3, 6, 16 and 24 h of culture). Bacterial smears on microscope slides were air dried, and poly-P granules were specifically stained by the procedure of Neisser^48^ with methylene blue/crystal violet and chrysoidin G as previously described^10^.

### Polyphosphate isolation and quantification

Analysis of poly-P accumulation in bacterial strains as a function of growth was performed by harvesting the bacteria and supernatants after growing the different strains in 15 ml of liquid MEI, LP-MEI or MRSc medium at 37 °C at different points of the growth curve (3, 6, 16 and 24 h). Poly-P was isolated by its resistance to hydrolysis with hypochlorite^49^ as previously described^10^. Briefly, bacterial cells were collected by centrifugation and lysed in 1 ml of 5% sodium hypochlorite. Insoluble material was pelleted by centrifugation at 16,000 × *g* and washed twice with 1 ml of 1.5 M NaCl/1 mM EDTA. Poly-P was extracted from the pellets with water, precipitated with NaCl and ethanol, and resuspended in 50 µl of water.

The extracellular contents of poly-P were determined as previously described^2^ by fluorescence using 4’,6’ diamino-2-fenilindol (DAPI) at a final concentration of 10 μM in 50 mM Tris–HCl pH 7.5, 50 mM NaCl buffer with an excitation wavelength of 415 nm and emission at 550 nm^50^ in a Clariostar fluorimeter (BMG LabTech). A sample of poly-P isolated from *B. longum* KABP042 was hydrolyzed with a volume of 2M HCl and incubation at 95 °C, 15 min, followed by neutralization by adding half volume of 2M NaOH. The released phosphate was measured with the Biomol Green kit (Enzo Life Sciences) and this quantified sample was used to build a standard curve for quantification using DAPI.

### Growth of the KABP042 strain in the presence of breast milk, HMO (lacto-N-tetraose; LNT) and polyamines

Optical density, pH, extracellular and intracellular contents of poly-P and expression of the *ppk* gene were determined for *B. longum* KABP042 at 6, 16 and 24 h of growth. The experiment was carried out in anaerobic jars, incubating the bacteria in 4 ml of MEI medium (control) or MEI medium in which glucose was replaced by 1% LNT or 10% (v/v) of a pool of four human milks and in MEI with a mixture of three polyamines at concentrations found in human milk (70.0, 424.2 and 610.0 10 nmol/dl for putrescine, spermidine and spermine, respectively^51^). Breast milk samples were obtained from 4 healthy women. The study was approved by the local ethics committee of Atención Primaria-Generalitat Valenciana (CEIC-APCV, registry 8108, approval 26/01/2017). All participants provided written informed consent and all experiments were performed in accordance with relevant guidelines and regulations.

### *ppk* gene expression

Four reference genes (16S rDNA, *tufA, rpoB*, and *atpD*^52^) were selected to normalize the expression of polyphosphate kinase target gene (*ppk*). Based on the *B. longum* JCM 1217 genome sequence (Genbank accession number AP010888), primer sequences were designed for *ppk* and the four reference genes in order to generate amplicons ranging from 50 to 100 bp in size (see Supplementary Table S2). Total RNA was isolated with TRIzol method as described previously^53^ from *B. longum* KABP042 grown in MEI or LP-MEI and in MEI with glucose replaced by LNT, MEI with glucose replaced by human milk and MEI plus a mixture of polyamines. RNA samples were treated with the Ambion Turbo DNA-free™ kit (Applied Biosystems) and first-strand cDNA was synthesized from 5 µg RNA using the SuperScript VILO cDNA synthesis kit (Invitrogen). Real-time qPCR was performed using the LightCycler 480 Real-Time PCR system (Roche Diagnostics, USA) with cDNA samples diluted 1/10 in duplicate and the LightCycler 480 SYBR Green I Master Mix (2X, Roche). The cycling conditions were as follows: 95 °C for 10 min, followed by 40 cycles of three steps consisting of denaturation at 95 °C for 10 s, primer annealing at 50 °C for 10 s, and primer extension at 72 °C for 20 s. Relative gene expression [R = (E_target_)^ΔCt target (mean control-mean sample)^/(E_ref_)^ΔCt ref (mean control-mean sample)^] was calculated with the tools implemented in the Relative Expression Software Tool (REST 2006, Qiagen), using bacteria grown in LP-MEI or MEI as the reference conditions, respectively.

### Tight junction protein gene expression

Caco-2 cells were seeded in 12-well plates at a density of 8 × 10^4^ cells/cm^2^, and after reaching confluence (10 days), they were exposed for 16 h to neutralized and diluted (1:10) supernatant medium of *B. longum* KABP042 cultured for 16 h in MEI and LP-MEI medium (see below). As control, cells were exposed to non-conditioned MEI and LP-MEI media at equal dilution. After exposure, the cells were recovered with 250 µl of TRIzol and RNA was extracted, quantified spectroscopically using a NanoDrop ND-1000 system (NanoDrop Technologies, USA) and treated with DNaseI. cDNA was obtained from 5 µg of total RNA using SuperScript VILO cDNA synthesis kit (Invitrogen). qPCR was performed using the LightCycler 480 Real-Time PCR system (Roche Diagnostics, USA). Reactions were carried out in a final volume of 10 µL containing 5 µL LightCycler 480 SYBR Green I Master Mix (2X, Roche), 1 µL cDNA (35 ng/µl), 1 µL of each forward and reverse primer (10 µM), and nuclease-free water. 18S ribosomal RNA and GADPH were employed as reference genes for normalization. The oligonucleotides employed are shown in Supplementary Table S1. The qPCR conditions were 95 °C for 5 min, followed by 40 cycles: 10 s denaturation at 95 °C, 10 s annealing at 55 °C, and 20 s elongation at 72 °C. Relative gene expression was calculated with the Relative Expression Software Tool. The reference conditions were those of cells cultivated in the presence of non-conditioned MEI or LP-MEI, respectively.

### Quantification of HSP27 expression

Quantification of HSP27 in the human colon carcinoma Caco-2 cells line was performed as described^2^. Briefly, *B. longum* KABP042 strain was grown for 16 h in MEI and LP-MEI medium and the supernatants (conditioned media) were obtained by centrifugation at 6000 X *g* for 10 min followed by filtration through 0.2 mm pore-size filters. Caco-2 cells were seeded at 8 × 10^4^ cells per well in 24-well culture plates with Minimum Essential Medium supplemented with 10% (v/v) fetal bovine serum and 1% (v/v) sodium pyruvate, 1% (v/v) non-essential amino acids, 10 mM HEPES, 0.0025 mg/L Fungizone and 100 U/ml Penicillin (cMEM) and incubated at 37 °C in an atmosphere with 95% relative humidity and 5% CO_2_, until confluence (10 days), with medium changes every 2–3 days. Bacterial supernatants (three independent cultures) were neutralized with NaOH solution and diluted 1:10 in cMEM medium. One milliliter aliquots of each diluted replicate were added to the cell cultures and incubation proceeded for 16 h. Cells from the wells were lysed by adding 50 µl of SDS-PAGE loading buffer per well and boiling prior separation in 12.5% SDS-PAGE gels. Proteins were transferred to Hybond ECL nylon membranes (GE Healthcare) and probed with rabbit polyclonal anti-HSP27 serum (Sigma) or with mouse monoclonal anti-β-actin antibody (Sigma).

### Caco-2 monolayer integrity

Integrity of Caco-2 cell monolayers was evaluated by measuring transepithelial electrical resistance (TEER) and the apparent permeability coefficients (*P*_app_) of the paracellular transport marker Lucifer Yellow (LY; Sigma). The assays were carried out in 12-well plates with polyester membrane inserts (pore size 0.4 μm, Transwell, Costar Corporation, Sigma). The cells were seeded (5 × 10^4^ cells/cm^2^) on the apical side to produce a cell monolayer. Then, 0.5 mL of cMEM was added to the apical chamber, and 1.5 ml of the same medium was added to the basolateral chamber. The cells were maintained in a humidified atmosphere of 5% CO_2_ and 95% air at 37 °C, with a change of medium every 2–3 days until differentiation (7–8 days post-seeding). During cell differentiation in the bicameral systems the TEER was measured with a Millicell-ERS voltohmmeter (Millipore Corporation) to evaluate the progress of the monolayers. Once the monolayer was formed and TEER measurements reached 300 Ohm/cm^2^, 0.5 ml of MEM medium containing 10% volume of supernatants from the growth of *B. longum* for 16 h in MEI or LP-MEI medium were added to the cell inserts. After 72 h of incubation, TEER and permeability to LY were determined as described^54^.

### Statistical analyses

All tests were performed in at least three independent cultures. The results were subjected to statistical analysis by one-factor analysis of variance (ANOVA) with Sidak post hoc multiple comparisons, two-factor ANOVA with Dunnett or Sidak post hoc multiple comparison, or by Student's *t*-test, according to design of each experiment (number of groups and presence or not of time factor), while correlation was assessed by Pearson test. All tests were performed with GraphPad Prism v8.0. Differences were considered significant at two-sided *p* < 0.05.

## Supplementary Information

Supplementary Figures.

Supplementary Tables.

## Data Availability

All data generated or analyzed during this study are included in this published article (and its Supplementary Information files) or are available upon request. All genomes investigated here were retrieved from the NCBI with accession numbers indicated in Table 2. For *B. bifidum* strain P671, the Whole Genome Shotgun project has been deposited at DDBJ/ENA/GenBank under the accession JAPFGE000000000. The version described in this paper is version JAPFGE010000000.
