# Supplementary material for: Study of the biosynthesis and functionality of polyphosphate in Bifidobacterium longum KABP042
Source: Sci Rep. 2023 Jul 8;13:11076. doi: 10.1038/s41598-023-38082-0 (PMC10329679; doi:10.1038/s41598-023-38082-0)
Supplement: Supplementary file 1 — Supplementary Figures. [file 41598_2023_38082_MOESM1_ESM.pptx]

## Slide 1
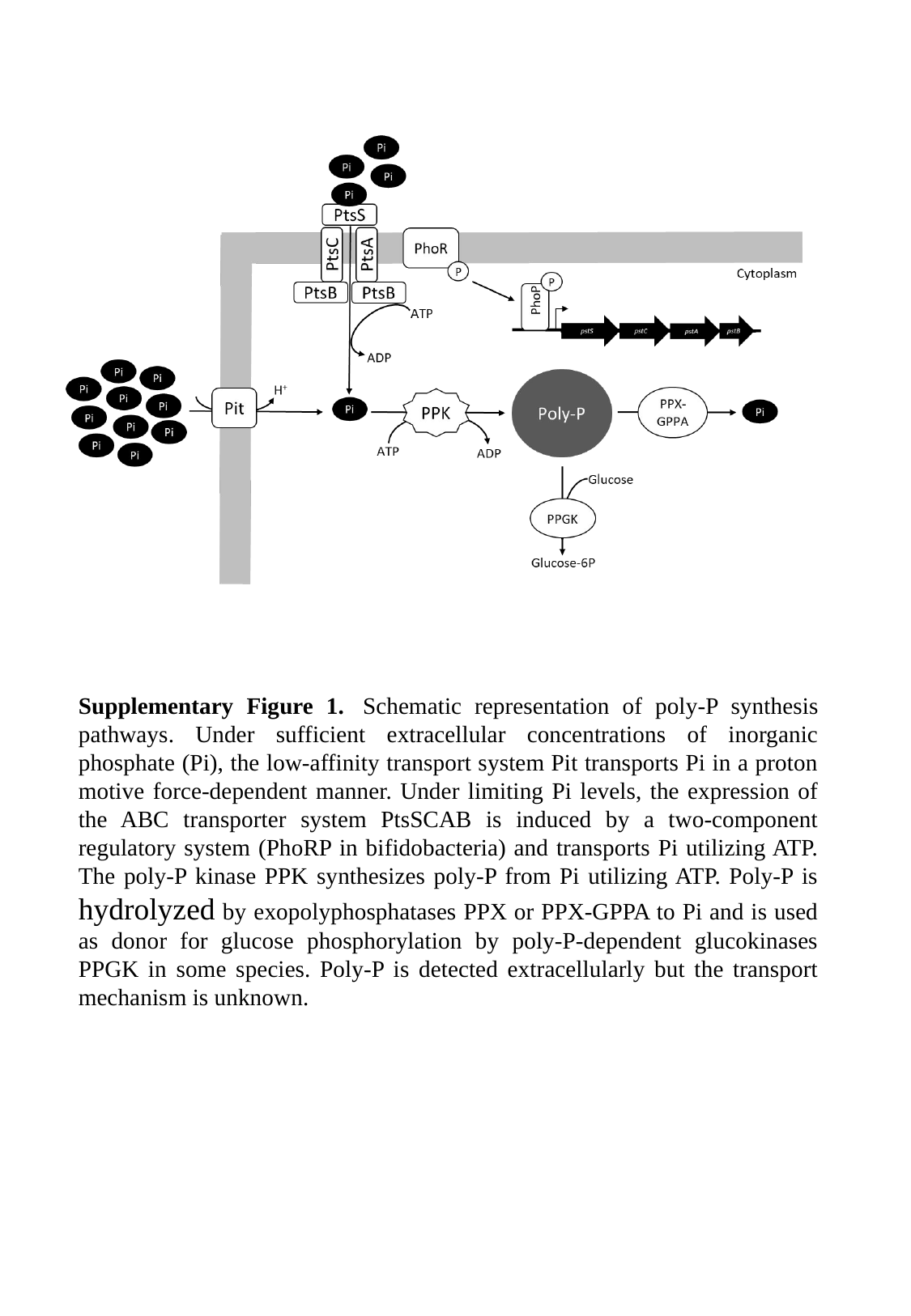

Supplementary Figure 1.  Schematic representation of poly-P synthesis pathways. Under sufficient extracellular concentrations of inorganic phosphate (Pi), the low-affinity transport system Pit transports Pi in a proton motive force-dependent manner. Under limiting Pi levels, the expression of the ABC transporter system PtsSCAB is induced by a two-component regulatory system (PhoRP in bifidobacteria) and transports Pi utilizing ATP. The poly-P kinase PPK synthesizes poly-P from Pi utilizing ATP. Poly-P is hydrolyzed by exopolyphosphatases PPX or PPX-GPPA to Pi and is used as donor for glucose phosphorylation by poly-P-dependent glucokinases PPGK in some species. Poly-P is detected extracellularly but the transport mechanism is unknown.

## Slide 2
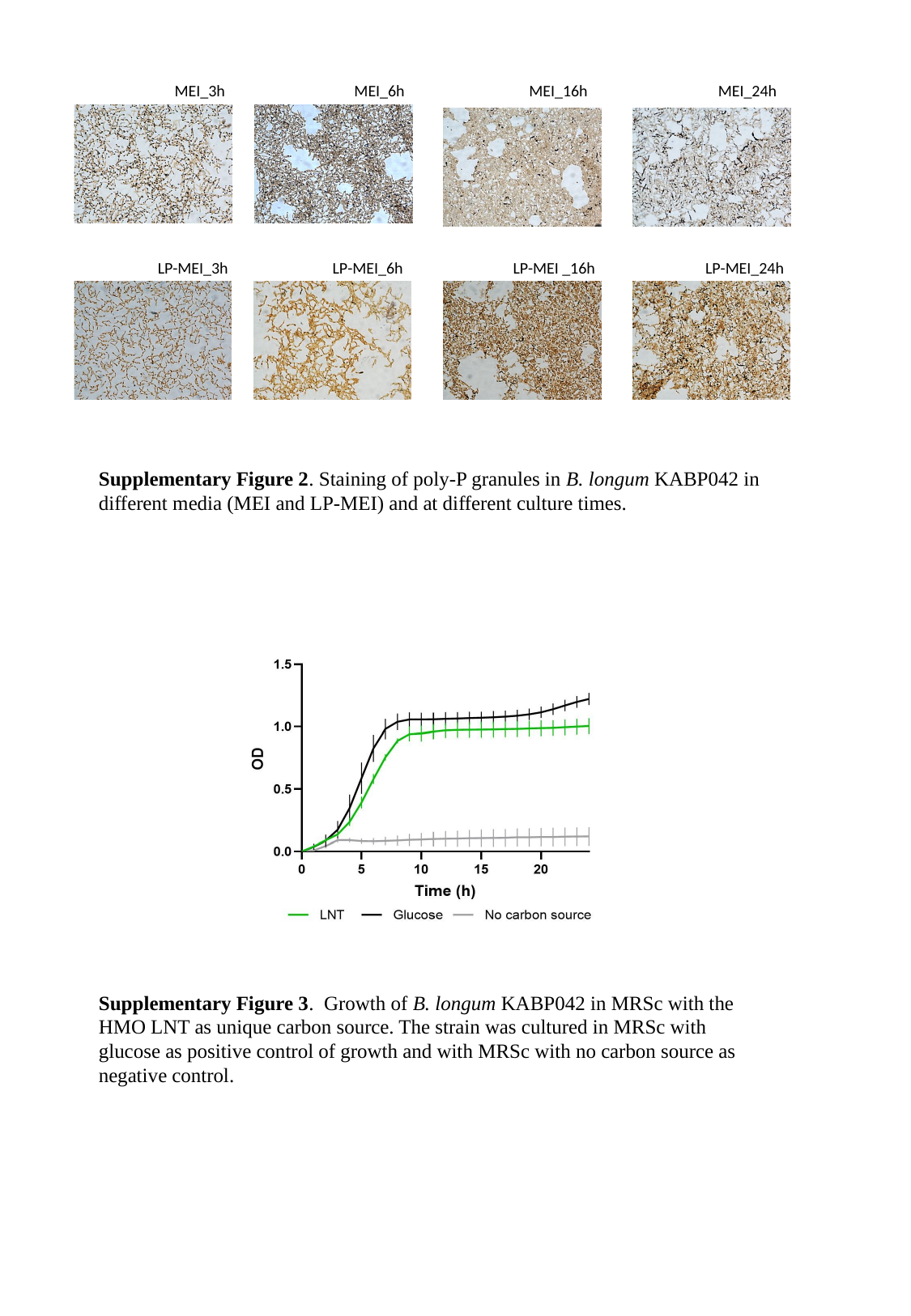

Supplementary Figure 2. Staining of poly-P granules in B. longum KABP042 in different media (MEI and LP-MEI) and at different culture times.
Supplementary Figure 3. Growth of B. longum KABP042 in MRSc with the HMO LNT as unique carbon source. The strain was cultured in MRSc with glucose as positive control of growth and with MRSc with no carbon source as negative control.

## Slide 3
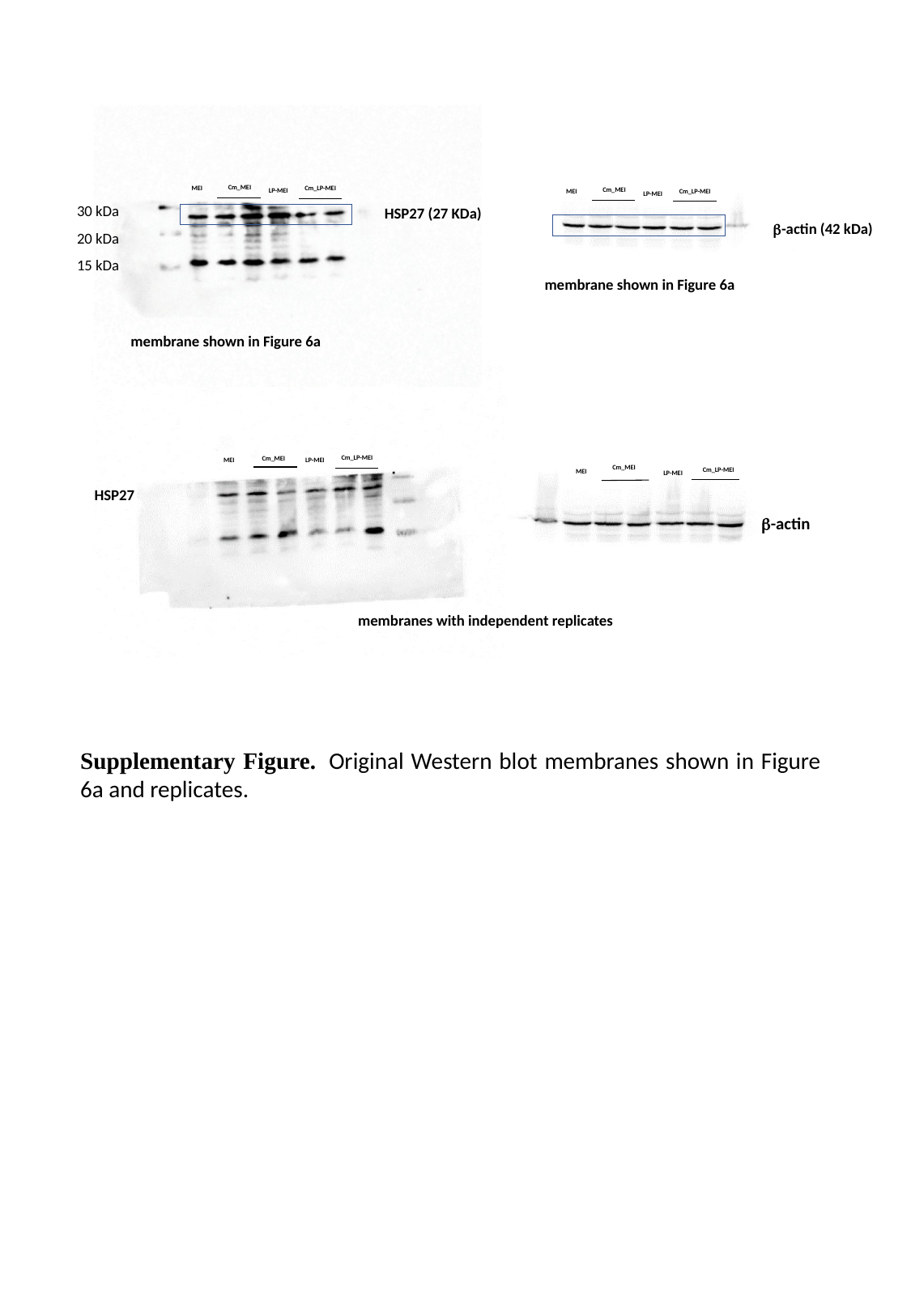

Cm_MEI
Cm_LP-MEI
MEI
LP-MEI
30 kDa
20 kDa
15 kDa
HSP27 (27 KDa)
membrane shown in Figure 6a
Cm_MEI
Cm_LP-MEI
MEI
LP-MEI
b-actin (42 kDa)
membrane shown in Figure 6a
Cm_LP-MEI
Cm_MEI
MEI
LP-MEI
HSP27
Cm_MEI
Cm_LP-MEI
MEI
LP-MEI
b-actin
membranes with independent replicates
Supplementary Figure.  Original Western blot membranes shown in Figure 6a and replicates.
