# Supplementary material for: Study of the biosynthesis and functionality of polyphosphate in Bifidobacterium longum KABP042
Source: Sci Rep. 2023 Jul 8;13:11076. doi: 10.1038/s41598-023-38082-0 (PMC10329679; doi:10.1038/s41598-023-38082-0)
Supplement: Supplementary file 2 — Supplementary Tables. [file 41598_2023_38082_MOESM2_ESM.docx]

**Supplementary information**

**Table S1.** Poly-P accumulation of studied strains after 6 and 16 h of growth in MEI or MRSc medium (mean ±SD). OD measured each time point is indicated.

| **Strain** | **Medium** | **6 h** | | **16 h** | |
| --- | --- | --- | --- | --- | --- |
|  |  | **nmol Pi/OD** | **OD** | **nmol Pi/OD** | **OD** |
| *B. animalis* BB12 | MEI | 5.75 ± 2.75 | 0.43 | 79.57 ± 33.59 | 2.46 |
|  | MRSc | 0.68 ± 0.33 | 1.85 | 0.07 ± 0.01 | 4.01 |
| *B. bifidum* P671 | MRSc | 0.24 ± 0.06 | 1.42 | 0.65 ± 0.13 | 4.01 |
| *B. breve* JCM1273 | MEI | 66.61 ± 3.64 | 1.05 | 38.12 ± 2.43 | 2.77 |
|  | MRSc | 20.82 ± 4.80 | 3.01 | 0.38 ± 0.11 | 7.91 |
| *B. breve* M16V | MRSc | 0.07 ± 0.03 | 2.22 | 4.52 ± 0.00 | 4.52 |
| *B. breve* P734 | MRSc | 6.75 ± 1.81 | 1.76 | 6.09 ± 0.50 | 3.61 |
| *B****.*** *longum* 35624 | MEI | 95.64 ± 17.23 | 2.08 | 24.52 ± 16.69 | 1.99 |
|  | MRSc | 49.05 ± 15.09 | 0.84 | 0.58 ± 0.01 | 3.93 |
| *B. longum* ATCC 15707 | MEI | 114.49 ± 7.52 | 1.48 | 1.52 ± 0.97 | 2.55 |
|  | MRSc | 87.85 ± 30.69 | 1.88 | 0.15 ± 0.01 | 1.97 |
| *B. longum* BB536 | MEI | 45.09 ± 12.42 | 1.36 | 1.35 ± 0.47 | 2.47 |
|  | MRSc | 1.29 ± 0.27 | 1.97 | 0.01 ± 0.01 | 4.99 |
| *B****.*** *longum* KABP042 | MEI | 186.17 ± 27.77 | 1.24 | 142.26 ± 2.70 | 1.82 |
|  | MRSc | 34.30 ± 2.33 | 2.36 | 14.01 ± 15.75 | 5.21 |
| *B. longum* P123 | MRSc | 10.45 ± 4.04 | 2.58 | 0.08 ± 0.01 | 5.49 |
| *B. scardovii* BAA-773 | MRSc | 0.50 ± 0.25 | 3.18 | 0.07 ± 0.02 | 8.23 |
| *L. paracasei* JCM1163 | MEI | 1.36 ± 0.71 | 4.63 | 0.29 ± 0.06 | 5.34 |
| *L. rhamnosus* GG | MEI | 0.90 ± 0.24 | 1.47 | 0.19 ± 0.03 | 3.69 |
| *L. plantarum* 299v | MEI | 0.08 ± 0.02 | 2.11 | 0.00 ± 0.00 | 3.70 |
| *L. plantarum* WCFS1 | MEI | 5.36 ± 5.28 | 2.38 | 0.00 ± 0.00 | 3.24 |
| *L. reuteri* DSM17938 | MEI | 0.15 ± 0.04 | 4.63 | 0.00 ± 0.00 | 5.34 |
| *P. pentosaceus* KABP041 | MEI | 0.09 ± 0.01 | 1.97 | 0.01 ± 0.01 | 3.47 |
| *S. boulardii* CNCM I-754 | MEI | 0.37 ± 0.01 | 1.19 | 0.15 ± 0.09 | 3.87 |

**Table S2.** Oligonucleotides used in this study

| oligonucleotide | Sequence |
| --- | --- |
| **Caco-2** |  |
| ZO1_F | GGAGTTGCAATGGTTAACGGA |
| ZO1_R | TCAGGATCAGGACGACTTACTGG |
| OCLN_F | AAGAGTTGACAGTCCCATGGCATAC |
| OCLN_R | ATCCACAGGCGAAGTTAATGGAAG |
| JAM_F | TGGCATTGGGCAGTGTTACAG |
| JAM_R | GTCTCCTTGGTCAAACTTCCAC |
| 18S_F | GTAACCCGTTGAACCCCATT |
| 18S_R | CCATCCAATCGGTAGTAGCG |
| GAPDH_F | CATGAGAAGTATGACAACAGCCT |
| GAPDH_R | AGTCCTTCCACGATACCAAAGT |
| ***B. longum* KABP042** |  |
| ppk_F | CACGTAGGCACCGGTAACTACA |
| ppk_R | AACAGACGAGTCAGATCCTGGC |
| rpoB_F | TGATCGTGGCGATGATGTCT |
| rpoB_R | TGTCGAGGCCGATTTCAAC |
| atpD_F | CGCCCACTTGGATGCAA |
| atpD_R | GATACCCTTGGAGGCAATGTCA |
| tufA_F | AAGGTGCTGCACGAGGAGTT |
| tufA_R | GAATCGATCTGGTTGAAGTCGTACT |
| 16S_F | GTGCCAGCMGCCGCGGTA |
| 16S_R | GCGTGGACTACCAGGGTATCT |
